# Genome-enabled phylogenetic and functional reconstruction of an araphid pennate diatom *Plagiostriata* sp. CCMP470, previously assigned as a radial centric diatom, and its bacterial commensal

**DOI:** 10.1038/s41598-020-65941-x

**Published:** 2020-06-10

**Authors:** Shinya Sato, Deepak Nanjappa, Richard G. Dorrell, Fabio Rocha Jimenez Vieira, Elena Kazamia, Leila Tirichine, Alaguraj Veluchamy, Roland Heilig, Jean-Marc Aury, Olivier Jaillon, Patrick Wincker, Zoltan Fussy, Miroslav Obornik, Sergio A. Muñoz-Gómez, David G. Mann, Chris Bowler, Adriana Zingone

**Affiliations:** 1 Fukui Prefectural University, Fukui, 917-0003 Japan; 2 Stazione Zoologica Anton Dohrn, Villa Comunale, 80121 Napoli, Italy; 3 Institut de Biologie de l’ENS (IBENS), Département de biologie, École normale supérieure, CNRS, INSERM, Université PSL, 75005 Paris, France; 4 Génomique Métabolique, Genoscope, Institut Francois Jacob, CEA, CNRS, Univ Evry, Université Paris-Saclay, 91057 Evry, France; 5 Biology Centre CAS, Institute of Parasitology, Ceske Budejovice, Czech Republic; 6 Charles University, Faculty of Science – BIOCEV, Prague, Czech Republic; 7 University of South Bohemia, Faculty of Science, Ceske Budejovice, Czech Republic; 8 Centre for Comparative Genomics and Evolutionary Bioinformatics, Department of Biochemistry and Molecular Biology, Dalhousie University, Halifax, Canada; 9 Royal Botanic Garden, Edinburgh, EH3 5LR Scotland UK; 10 Institute for Food and Agricultural Research and Technology (IRTA), E-43540 Sant Carles de la Ràpita, Catalunya, Spain; 11 Present Address: Stony Brook University, School of Marine and Atmospheric Sciences, Southampton New York, USA; 12 Present Address: Université de Nantes, CNRS, UFIP, UMR 6286, F-44000 Nantes, France

**Keywords:** Ecology, Microbiology

## Abstract

Diatoms are an ecologically fundamental and highly diverse group of algae, dominating marine primary production in both open-water and coastal communities. The diatoms include both centric species, which may have radial or polar symmetry, and the pennates, which include raphid and araphid species and arose within the centric lineage. Here, we use combined microscopic and molecular information to reclassify a diatom strain CCMP470, previously annotated as a radial centric species related to *Leptocylindrus danicus*, as an araphid pennate species in the staurosiroid lineage, within the genus *Plagiostriata*. CCMP470 shares key ultrastructural features with *Plagiostriata* taxa, such as the presence of a sternum with parallel striae, and the presence of a highly reduced labiate process on its valve; and this evolutionary position is robustly supported by multigene phylogenetic analysis. We additionally present a draft genome of CCMP470, which is the first genome available for a staurosiroid lineage. 270 Pfams (19%) found in the CCMP470 genome are not known in other diatom genomes, which otherwise does not hold big novelties compared to genomes of non-staurosiroid diatoms. Notably, our DNA library contains the genome of a bacterium within the Rhodobacterales, an alpha-proteobacterial lineage known frequently to associate with algae. We demonstrate the presence of commensal alpha-proteobacterial sequences in other published algal genome and transcriptome datasets, which may indicate widespread and persistent co-occurrence.

## Introduction

The diatoms are important primary producers in aquatic ecosystems, being responsible for 20% of global net primary productivity^1^, and play an important role in the biological carbon pump^2^. Diatoms are proposed to number within the tens of thousands of species^3^, as has been further supported by analysis of the *Tara* Oceans dataset^4^. The evolutionary diversity of diatoms has been studied extensively: historically by means of morphological and fossil information, and recently also through molecular phylogenetics. Recent phylogenetic studies, using multigene datasets, have consistently recovered the following results: (1) among the ancestral “centric” diatoms, radial centrics are paraphyletic to the polar centric lineages, (2) the polar centric diatoms are themselves paraphyletic to the monophyletic “pennate diatoms”, and (3) the pennate clade comprises araphid species, which are paraphyletic, and raphid species, which are monophyletic^5,6^. Phylogenetic studies of diatoms now routinely use multigene markers, often in a combined dataset with small subunit ribosomal DNA (SSU), the Rubisco large subunit (*rbc*L), and the photosystem II binding complex (*psb*C), to reconstruct higher level phylogeny (e.g., above genus to class relationships)^7^.

Well-curated culture collections are an ideal resource for phylogenetic studies when one tries to characterize a particular group of organisms, as strains are often tied to various useful information such as sampling sites and dates, suitable culture conditions, and sometimes microscopic images and even gene sequences^8,9^. In the course of previous projects aiming to reveal the diversity of leptocylindrids (e.g.^10–13^), we obtained the strain CCMP470, which was previously annotated as *Leptocylindrus danicus*, from the National Center for Marine Algae and Microbiota (NCMA, formerly CCMP). Our preliminary analysis, however, clearly indicated the position of this strain as an araphid pennate diatom, despite its apparent centric morphology. To further characterize this strain, we have reconstructed its morphology, molecular phylogeny, and assembled a draft genome. Based on an extensive number of sequences corresponding to a rhodobacteralean symbiont, we have also investigated potential symbiotic bacterial interactions in other cultured algal lineages.

## Results and Discussion

### Morphological characterization of CCMP470

**Figure 1:**
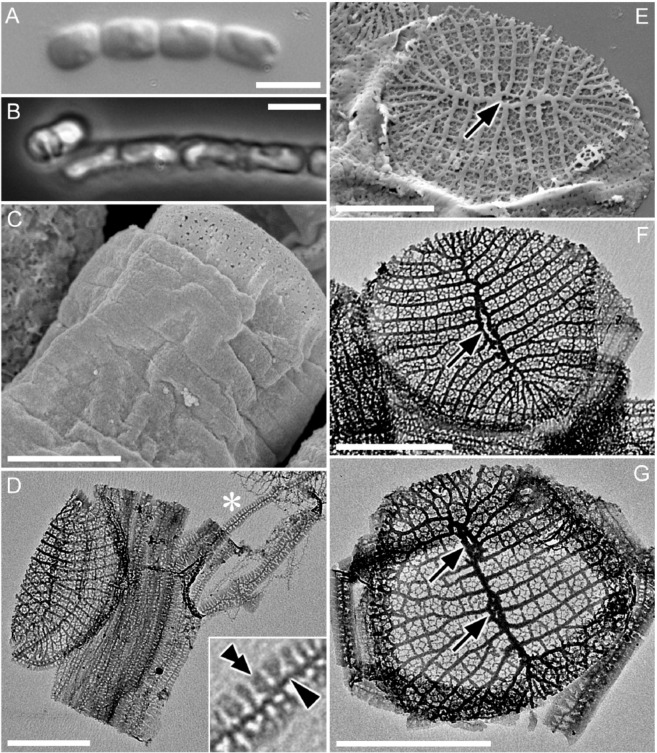
Morphology of CCMP470 under LM (**A**,**B**), SEM (**C**,**E**) and TEM (**D**,**F**,**G**). (**A**) Chain colony under bright field, and (**B**) under phase contrast. (**C**) Frustule in oblique view, showing numerous girdle bands. Notice the slightly wrinkled appearance of the bands even after preparation by critical-point drying, indicating weak silicification of frustule. (**D**) Collapsed theca with >10 girdle bands. Each band has a primary rib from which secondary ribs extend perpendicularly. The inset is an enlargement of the part marked by an asterisk, showing the primary and secondary ribs of a band (arrowhead and double arrowhead, respectively). (**E–G**) Circular valve with a distinct sternum. Virgae extend perpendicularly from the sternum but become radiate towards the periphery of the valve. Occasional bifurcation takes place to keep the stria density even throughout valve face (indicated by arrows), except for valve apices which display a distinct region with slightly finer striae; these most likely represent apical fields responsible for mucilage secretion. The highly reduced labiate process formed along the sternum can be inconspicuous (**E**), transapically elongated (**F**), or double (**G**). Scales = 5 µm (**A**,**B**) and 1 µm (**C**–**G**).

We characterized the ultrastructure of CCMP470 using light and electron microscopy (LM and EM, respectively). Cells were cylindrical or somewhat barrel-shaped, and were attached to one another by their valve faces to form a chain colony (Fig. 1A,B). A single plastid was recognizable in phase contrast microscopy images (Fig. 1B). The frustules were weakly silicified, as was evident from the observation that critical-point-dried specimens showed a wrinkled surface, particularly in the girdle region (Fig. 1C). Under LM, the width of the lid/bottom of the barrel-shaped cell, which likely represented the diameter of the circular valve, was 2.0 ± 0.7 (1.2–3.4) μm (n = 15). Acid cleaned materials were air-dried, resulting in the collapse of the frustule’s three dimensional structure (Fig. 1D–G), which further confirmed their weak silicification. Girdle bands were numerous (Fig. 1C,D). Although the band surface appeared to be plain in critical-point-dried material observed under SEM, pores were evident in acid-cleaned material observed with TEM (Fig. 1D,F,G). Each band comprised a primary rib running along its long axis (e.g., Figure 1D, arrowhead), from which secondary ribs further extended transversely in both advalvar and abvalvar directions (Fig. 1D, double arrowhead). The secondary ribs were regularly spaced at their bases, resulting in a somewhat regular areolation pattern, but were not fused at the margins (Fig. 1D). The valve had a circular to oblong outline. A sternum ran roughly across its long axis, occasionally showing undulation and/or bifurcation and fusion, which ended up with the formation of an annular structure mostly close to the centre of the valve (Fig. 1E,F, arrow). We interpret this annular structure as a highly reduced labiate process, based on its more or less central position, as well as the fact that its phylogenetic relatives also possess a reduced labiate process in a similar position (see below). We observed valves with more than one annulus along the sternum (Fig. 1G, arrows), although this type of valve can be considered as exceptional, due to its low frequency in the sample. Virgae extended perpendicularly from the sternum, and vimines cross-linked the virgae to form ± square areolae.

In LM, the cylindrical cells, attached to one another by their valve faces to form chains, resemble the radial centric genus *Leptocylindrus*, which explains the former annotation of this strain as *Leptocylindrus danicus* in the NCMA culture collection. However, the ultrastructure is instead consistent with the staurosiroid clade of araphid pennate diatoms. These are small-celled araphids^14–16^ characterized by the presence of a distinct sternum with parallel striae, features considered as synapomorphies of pennates^14,15^, and the absence in general of a labiate process on the valve^14^ (Fig. 1). However, these features are not universally conserved across staurosiroids. The genus *Plagiostriata*, for example, has an atypical labiate process with an apparently reduced architecture, with no stalk-like structure at its base. Rather, it takes the form of a penetration on the valve face with a distinct marginal area (figs. 14, 15 in^15^, figs. 330–339 in^16^). It should be noted in addition that the labiate process in *P. goreensis* is located at the middle of one side of the sternum, in a position similar to that of the annulus observed in CCMP470 (Fig. 1E,F). There are also some non-staurosiroid araphid diatom genera possessing structurally “modest” labiate processes (i.e., with less elevated architecture and no stalk-like structure at the base) at the centre of the valve, such as *Diatoma* and *Tabellaria*^17,18^. An alternative interpretation for the CCMP470 annulus is that it is an incompletely fused sternum. The pattern centre of the valve in araphid diatoms may perhaps be an elongated annulus that usually gives rise to a sternum by infilling during further valve morphogenesis^19^. However, because valves with annular structure were dominant throughout the observations using many asynchronous cultures fixed at different times, it is unlikely that they are only present during valve formation. Therefore, the lightly silicified valves of CCMP470 observed in this study were probably mature, leaving the annular part unmasked or incompletely fused. Superficially similar valves can also be seen in chain-forming planktonic centric diatom species such as *Subsilicea* and *Papiliocellulus*^20^.

### Draft genome and phylogeny

**Figure 2:**
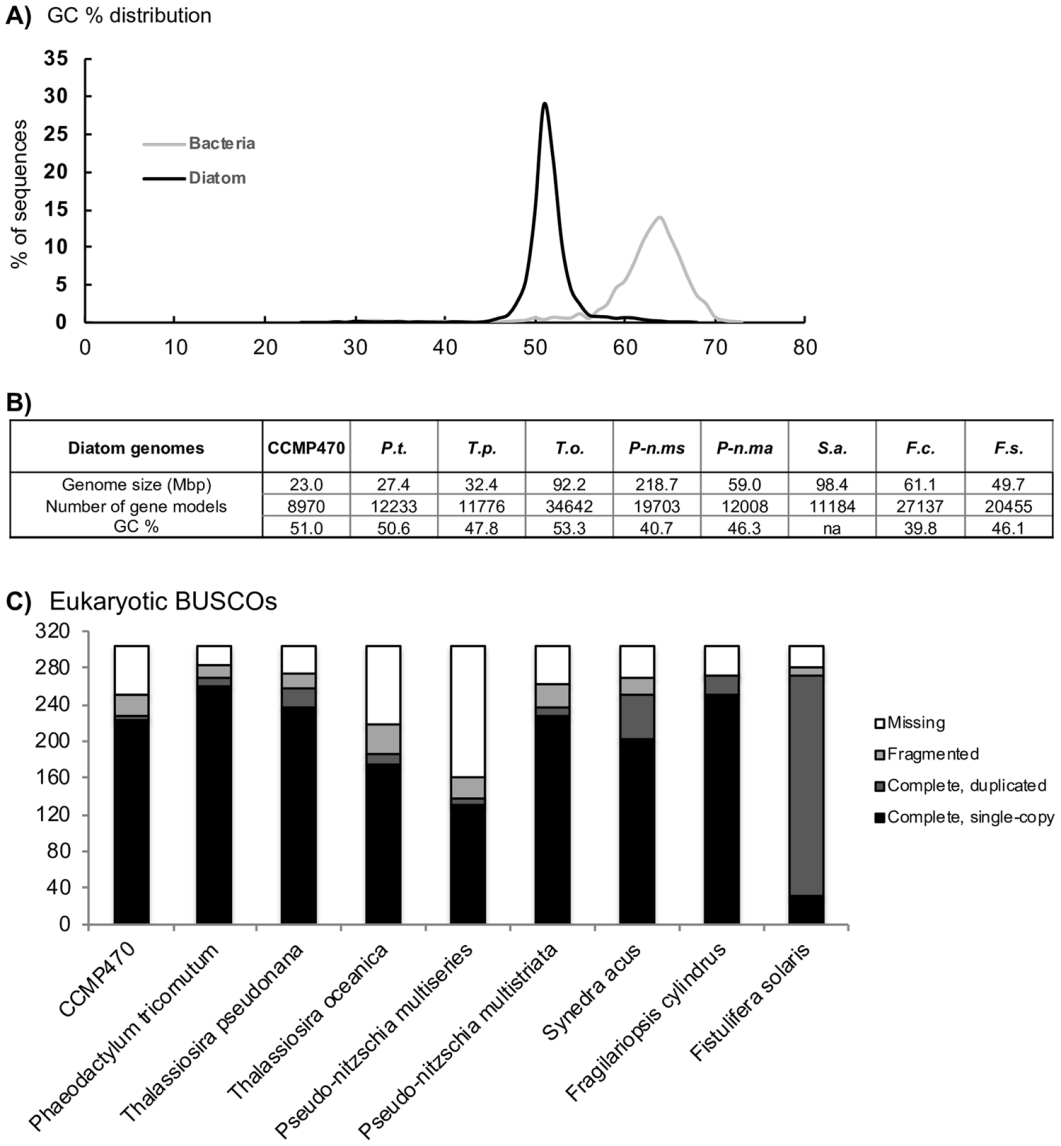
Features of the CCMP470 genome (**A**) GC content plots of the CCMP470 genome and that of its bacterial symbiont. (**B**) Comparisons of key features of the CCMP470 genome with those of other diatom species (*P.t., Phaeodactylum tricornutum; T.p., Thalassiosira pseudonana; T.o., T. oceanica; P-n.ms, Pseudo-nitzschia multiseries; P-n.ma, Pseudo-nitzschia multistriata; S.a., Synedra acus; F.c., Fragilariopsis cylindrus; F.s., Fistulifera solaris*), assembled from^24,25,30–32,66^. “na” indicates the corresponding information was not recorded in the genome release. (**C**) BUSCO version 3 coverage of different diatom genomes assessed using eukaryotic gene models^26^; equivalent outputs using plant and protist gene models are shown in Fig. S1B and Table S1.

To better understand the evolutionary placement of CCMP470, we sequenced and assembled a draft genome. The crude CCMP470 genome sequence was separated into two constituent sets of contigs with differing GC contents, with medians of 64% and 51%, respectively (Fig. 2A; Table S1), indicating that it in fact represents a simple metagenome. These contigs were confirmed using MetaBAT^21^ and last common ancestor (LCA) reconstruction analysis^22,23^ to correspond to two, different co-sequenced organisms: the diatom host and a bacterial symbiont (Fig. S1Aand Dataset S1). The CCMP470 host genome assembly is 22.99 Mb and consists of 4,992 contigs, containing 8,970 gene models, slightly fewer than the numbers (11,184–34,500) annotated in other diatom genomes (Fig. 2B). BUSCO analysis revealed that the CCMP470 genome was of an equivalent level of completeness (250/303 eukaryotic BUSCOs identified) to the genomes of other diatom species, and was substantially more complete than genome sequences from *Thalassiosira oceanica, Pseudo-nitzschia multiseries,* and to some extent *Synedra acus*^24,25^ (Fig. 2C; S1B). The BUSCO analysis further suggested that there were very few gene duplications in the CCMP470 genome (Fig. 2C).

**Figure 3:**
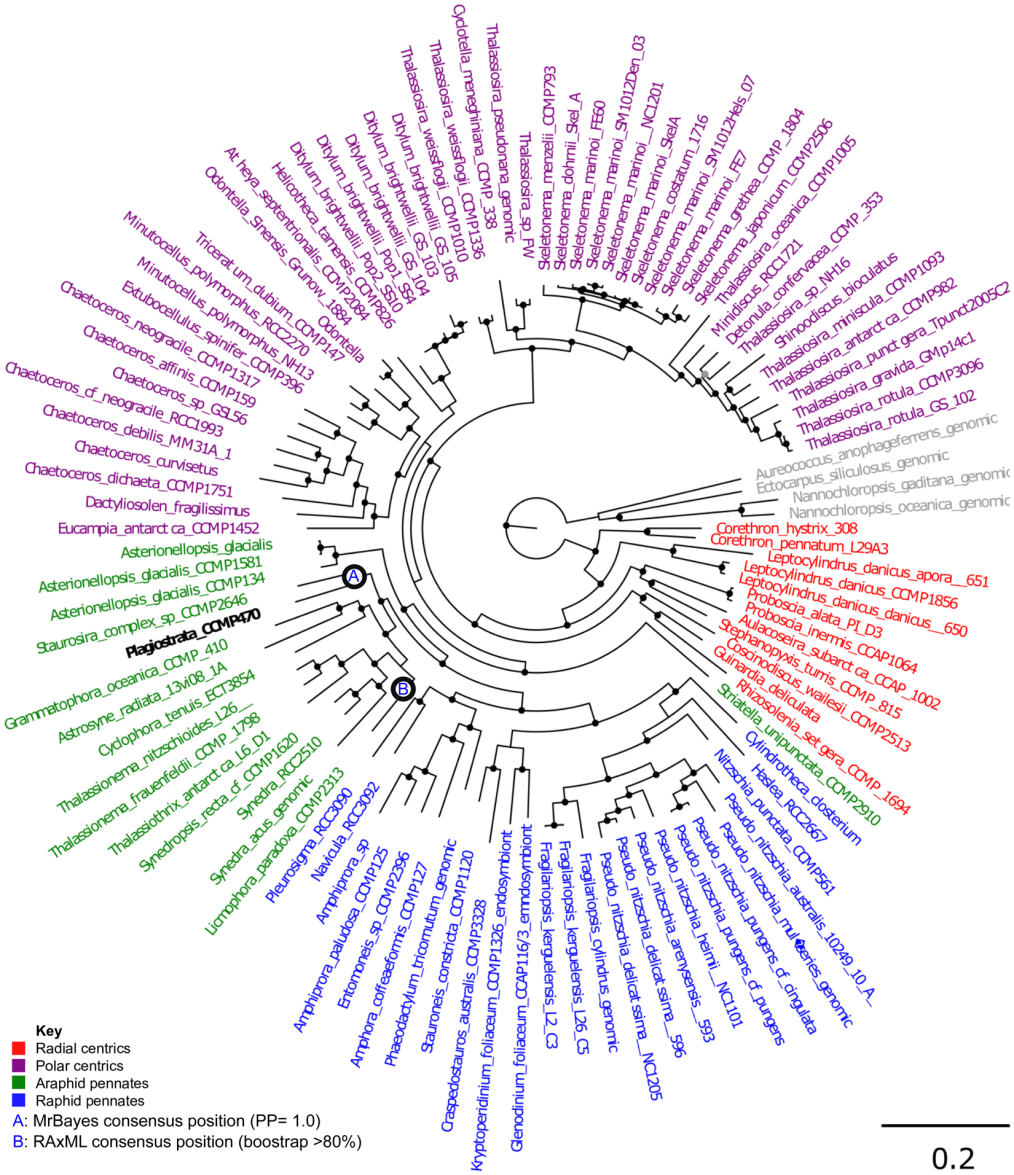
Evolutionary position of CCMP470 based on BUSCO sequences. A. Consensus topology of a 106 taxa × 16,774 aa alignment of 35 BUSCOs conserved in published diatom genome and transcriptome libraries, inferred with MrBayes and RAxML using three substitution matrices (MrBayes: GTR, Jones, WAG; RAxML: GTR, JTT, WAG). The tree topology is rooted on four evolutionary outgroup ochrophyte genomes (in grey). Diatom taxa are shaded by evolutionary origin, and *Plagiostriata* CCMP470 is shown in black. The topology shown is the consensus MrBayes topology; alternative phylogenetic positions for CCMP470, as inferred using Bayesian and RAxML analysis, are shown with labelled circles.

A concatenated multigene tree was built using a 65 taxa × 16,774 amino acid alignment of 35 eukaryotic BUSCOs identified to have widespread conservation (present in complete or fragmented form in >60% sampled species, average copy number per species <1.5) across all diatoms, following previously established methodology^26,27^ (Table S2). This topology strongly supported placement of CCMP470 within the araphid pennate diatoms, with a sister-group position to *Staurosira* robustly supported (posterior probability 1.0) in all three MrBayes trees generated with the concatenated library, and 15 of the 33 single-gene RAxML trees in which it was included (Fig. 3; Table S2).

**Figure 4:**
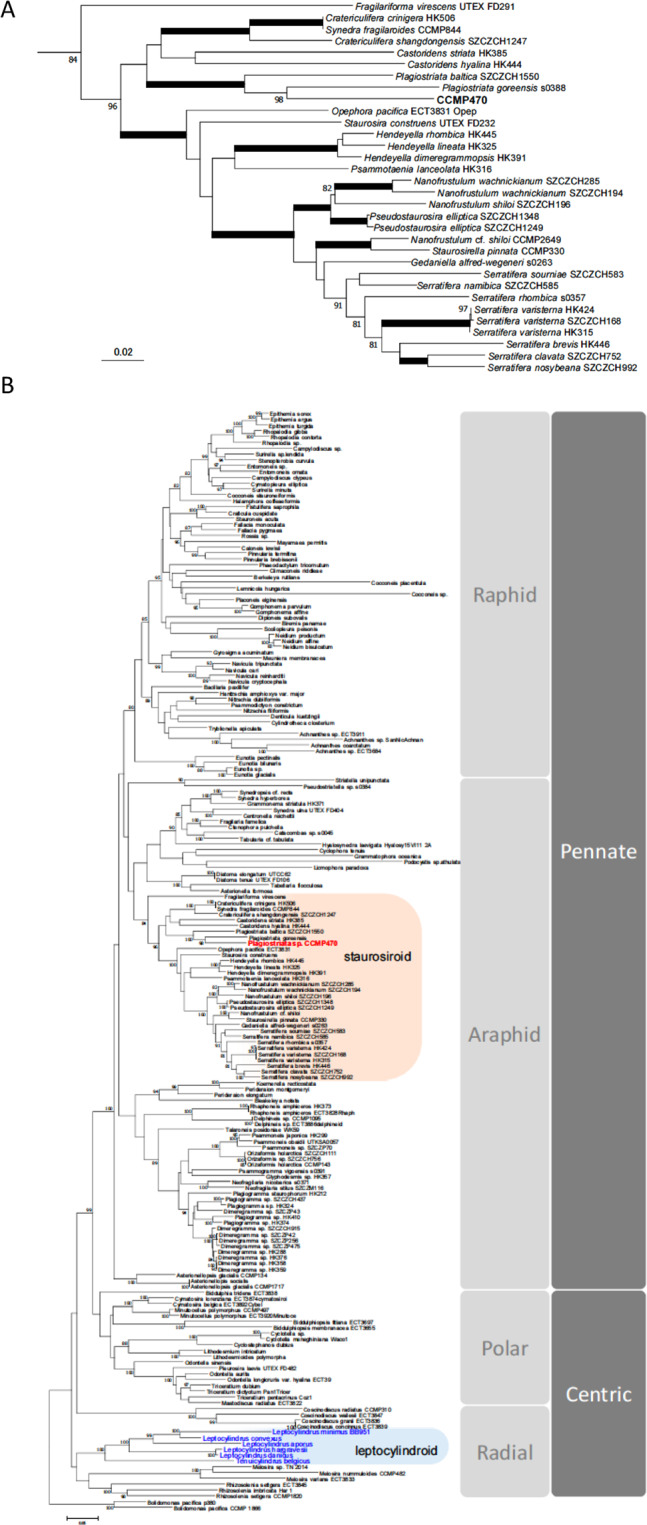
Evolutionary position of CCMP470 based on SSU, *rbc*L and *psb*C sequences. (**A**) Enlarged view of the staurosiroid clade based on a combined dataset of SSU, *rbc*L and *psb*C. Nodes with thicker lines indicate 100% bootstrap value. For simplicity only strong nodal support (bootstrap value >80%) is shown. Scale = 0.05 substitutions/site. (**B**) Complete topology inferred for alignment in A), including non-staurosiroid taxa.

We also generated a more species-rich phylogenetic tree using a concatenated alignment of SSU, *rbcL* and *psbC* produced by Theriot and colleagues^7^ but with more leptocylindrid and staurosiroid taxa sequenced^11,13,14,16^, in order to infer the phylogenetic position of CCMP470 with higher resolution (Fig. 4; Table S2). Within the staurosiroid clade, *Fragilariforma virescens* UTEX FD291 formed a sister-group to all other lineages, with moderate bootstrap support (BS: 84). The rest of the members bifurcated (with BS: 96) into two clades. One with the highest support, comprised *Opephora, Staurosira, Hendeyella, Psammotaenia, Pseudostaurosira, Nanofrustulum, Staurosirella* and *Serratifera*, and the other, with no nodal support (bs: <70), contained *Synedra, Cratericulifera, Castoridens* and *Plagiostriata*, in which CCMP470 and two *Plagiostriata* strains (*P. baltica* SZCZCH1550 and *P. goreensis* s0388) formed a robust clade, CCMP470 being nested within the *Plagiostriata* species.

### Functional and evolutionary characterization

**Figure 5:**
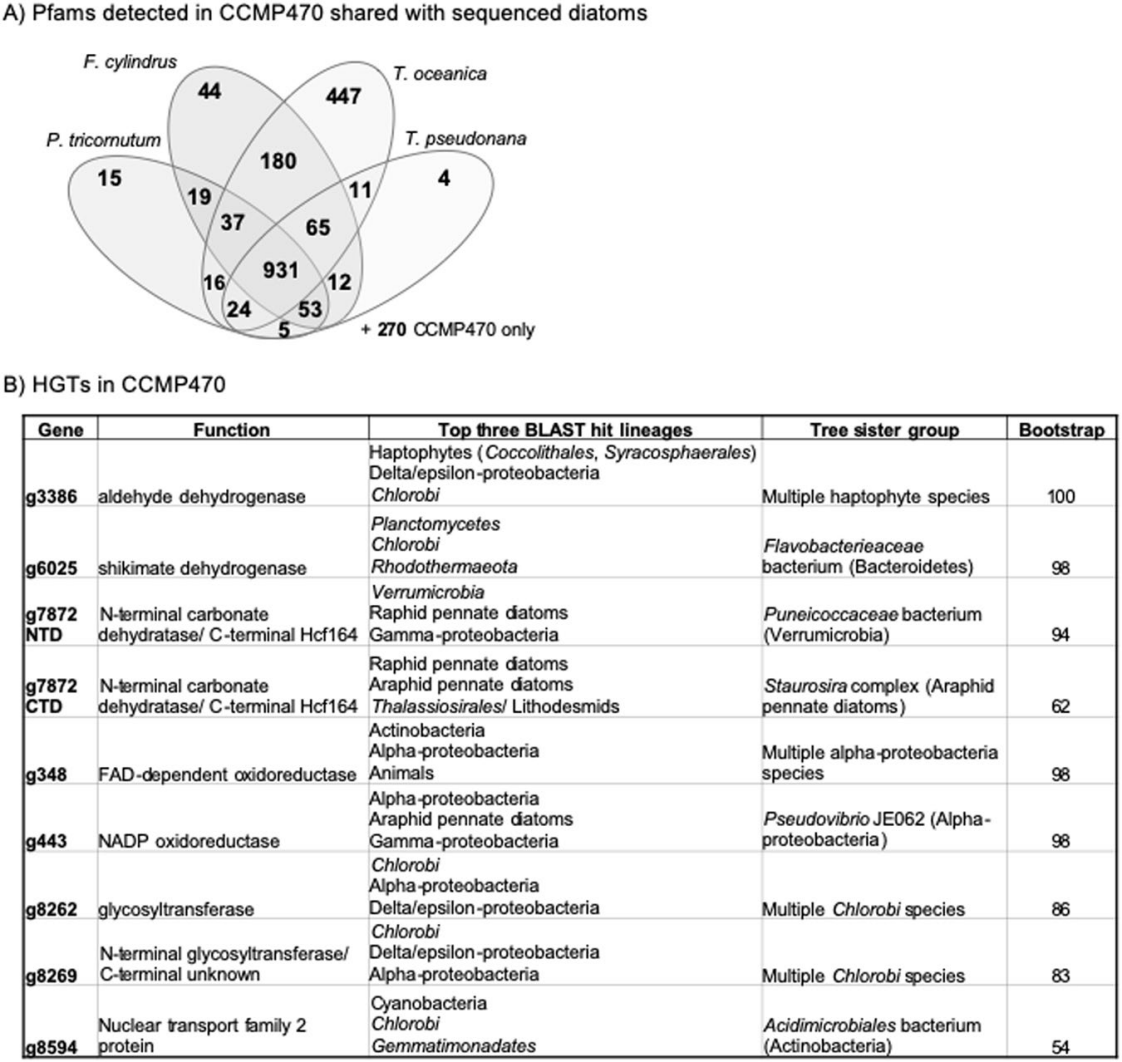
Functional and evolutionary profiling of the CCMP470 genome. (**A**) Combinations of Pfams found in other sequenced diatom genomes from CCMP470 host genome contigs. 270 Pfams were found uniquely in CCMP470 (shown outside the Venn diagram). GO enrichment data, and specific Pfam domains associated with CCMP470 and not with other diatoms, are shown in Fig. S2B. Individual Pfams for each gene in the CCMP470 genome are provided in Table S1, in the sheet labelled “function”. Tabulated totals of each Pfam for CCMP470, and for each other reference diatom genome, are provided in Table S1 in the sheet labelled “PFAMs”. (**B**) Evolutionary origins of eight genes verified through both BLAST rank and single-gene tree approaches to have been acquired via horizontal gene transfer into the CCMP470 genome. Exemplar trees and structural diagrams for one protein (g7872) inferred to be a chimera of bacterial and diatom origin are shown in Fig. S4.

We wished to determine what functions are encoded in the CCMP470 genome. GO category annotation was generated using protein families (Pfams) found by CLADE^28^ and refined by GO consortium PFAM2GO^29^. We found the genome to be enriched in genes encoding functions associated with organelle biogenesis (Golgi apparatus, ER membrane, chloroplast, nuclear chromosome; Fig. S2A). We also identified 270 Pfams that are encoded within this genome, but not found in the genomes of the diatoms *Phaeodactylum tricornutum, Thalassiosira pseudonana, T. oceanica*, or *Fragilariopsis cylindrus* (Fig. 5A)^24,30–32^. These include two tetratricopeptide domains (PF07926, PF13429) and a methanol dehydrogenase (PF13360), each found in >5 copies in the CCMP470 genome, but absent from all four remaining diatoms (Fig. S2B). Conversely, we identified 358 Pfams found in all four remaining diatom genomes, but absent from CCMP470, including a pentatricopeptide domain (PF01535) and zinc-binding alcohol dehydrogenase (PF00107), both present in >5 copies in all other diatoms (Fig. S2B).

Next, we investigated whether any genes in the CCMP470 genome have specific evolutionary ancestries that separate them from other diatom lineages. The evolutionary origin of each gene in the CCMP470 genome was investigated using a reciprocal BLAST best hit (RbH) search, integrating published genomic and transcriptomic data from across the tree of life, and divided into 144 sub-categories based on recently published taxonomies^5,23,27^ (Table S3). Consistent with the phylogenetic placement, the single diatom taxonomic category with the most reciprocal BLAST best-hits was the araphid pennate diatoms (7,977 genes with RbH matches), followed by raphid pennate species (7,819 genes), with a smaller number of RbH matches (5,974 genes) obtained for *Leptocylindrus* (Table S3).

We used the outputs of the RbH analyses to search for genes that uniquely possess orthologues in close relatives, following taxonomic nomenclature established by Dorrell and colleagues in^33^. Through this, we identified five genes that only produced BLAST hits with expect value below 10^−05^ in other araphid pennate libraries (principally *Staurosira complex*; Fig. S3A), and thus presumably originated within this lineage. Although none of these genes contained known protein domains, alignments with their homologues revealed large numbers of identical residues, indicating probable functional conservation (Fig. S3B).

We additionally used a previously published phylogenomic pipeline, based on ranking the reciprocal BLAST top hits from each of the 144 sub-categories through BLAST search, followed by single-gene trees, to identify evidence of horizontal gene transfer into a recent ancestor of CCMP470 (Table S4)^34^. The majority of the genes identified were either most closely related to other diatom sequences, or could not retrieve clear non-diatom origins through the BLAST top hit analysis; or were found to be too divergent to align with their respective BLAST top hits to allow phylogenetic inference. However, we could confidently identify eight candidate HGT events (resolving with a sister group corresponding to the BLAST top hit taxon, with RAxML bootstrap support >50%; Fig. 5B). Seven of the HGT genes are of apparent prokaryotic (chlorobial, alphaproteobacterial, and verrucomicrobial) origin; and the remaining HGT fell in an unresolved position within the haptophytes (Fig. 5B). Seven of the HGT genes occur on contigs containing at least one sequence of verified diatom origin, indicating that they are not contaminants within the diatom sequence fraction (Table S4). The remaining HGT gene (g7872 C) is an apparent chimeric or “S-gene”^34^, consisting of an N-terminal carbonate dehydrogenase domain of apparent verrucomicrobial origin; and a C-terminal Hcf164 domain of diatom origin (Fig. S4).

### Origin and function of the CCMP470 symbiont

Next, we considered the genome of the CCMP470 bacterial symbiont, which was 3.48 Mb in size and consisted of 3,158 genes (Table 1A; Table S1 and Dataset S1). The majority of the bacterial contigs were identified using LCA annotations as resolving within the Rhodobacteraceae, an alpha-proteobacterial group frequently identified as algal symbionts and associated with algal blooms^35–39^. We used a set of 27 genes from a list of 40 previously identified as single-copy phylogenetic markers for alpha-proteobacteria^40^, that could be detected on the symbiont contigs (Table S2), to further identify sequences of probable alpha-proteobacterial commensal origin in six other published algal genomes and transcriptomes^41,42^ (Table 1B).

**Table 1:** Characterization of the CCMP470 symbiont.

A. CCMP470 bacterial commensal genome		
Number of contigs		1298
Number of gene models		3158
GC%		61%
**B. Species in which alpha-proteobacterial commensals detected**		
*Plagiostriata CCMP470*	Araphid pennate diatoms	genomic
*Asterionellopsis glacialis CCMP134*	Araphid pennate diatoms	MMETSP transcriptome
*Cladosiphon okamuransis*	Phaeophytes	genomic
*Bathycoccus prasinos*	Prasinophytes	1KP transcriptome
*Chaetoceros curvisetus*	Polar centric diatoms	MMETSP transcriptome
*Nannochloropsis oculata*	Eustigmatophytes	1KP transcriptome
*Mantoniella squamata*	Prasinophytes	1KP transcriptome

A. Summary of the bacterial commensal sequence that is part of the CCMP470 genome. B. Tabulated algal genomes and transcriptomes in which at least two of a set of 27 marker genes^40^, of phylogenetically verified alpha-proteobacterial origin, could be identified. See Materials and Methods for more details.

We also performed reciprocal BLAST best hit and BLAST rank analyses of the symbiont genome, as we had done for the diatom, to identify genes that might have arisen in the bacteria via recent horizontal gene transfer (Fig. S5A). We identified two genes likely to represent genuine HGT events by single-gene trees, arising, respectively, from gamma-proteobacterial and verrucomicrobial donors, the first of unknown function and the second belonging to the abortive infection phage resistance (AIPR) protein family (Table S4). We noted a small number of genes that, based on BLAST rank analyses, had closest evolutionary relatives amongst diatoms and other stramenopile lineages (Fig. S5B). Single-gene phylogenies indicate that these genes frequently have alpha-proteobacterial second sister groups, reinforcing the presence of bacterial symbionts within these cultures (Table S4).

### *In silico* analysis of metabolic interactions between CCMP470 and its bacterial symbiont

Since previous studies have shown that CCMP470 can form symbiotic interactions for nutrient exchange with Rhodobacteraceae, e.g., supplying fixed organic carbon to the species *Planktotalea frisia*^43^, we explored the potential of metabolite exchange between CCMP470 and its co-sequenced symbiont *in silico*. We searched for possible metabolic interactions between the two species using ModelSEED^44^. This identified 1,201 reactions based on 768 annotated genes for the bacterium, and 1,084 reactions from 502 genes for the diatom (Table S5 and Dataset S2). We noted that the bacterial commensal encodes the complete pathway for the synthesis of bacteriochlorophyll, from geranylgeraniol and chlorophyllide precursors, suggesting that it is likely to be photo-heterotrophic. Previous studies of algal-bacterial symbioses have experimentally evidenced B-vitamins as mediators of symbiotic interactions, e.g., in the model laboratory system involving *Lobomonas rostrata* and *Mesorhizobium loti*^45^, and the marine species *Ostreococcus tauri* and *Dinoroseobacter shibae*^46^. We found no evidence of B-vitamin auxotrophies in either CCMP470 or its symbiont, as both organisms encode complete pathways for thiamin, niacin, folate and biotin biosynthesis. CCMP470 is unlikely to require cobalamin (vitamin B_12_) obligately, as it encodes a putative cobalamin-independent methionine synthase (METE, 5 methyl-tetra-hydropteroyl-triglutamate-homocysteine methyltransferase, g2483.t1)^47^; and indeed we could not identify a complete cobalamin biosynthesis pathway in the symbiont. We did note that the symbiont retains a gene encoding BluB, which adds the DMB moiety to pseudocobalamin, forming cobalamin, which would imply that the species is able to use B_12_ if it can acquire intermediates from its environment^48^. The ModelSEED analysis predicted that the symbiont is able to process dehydroepiandrosterone sulfate (a sulfate ester), and a range of sulfite and sulfate compounds; and previous studies have shown that certain Rhodobacteraceae can metabolize organosulfur compounds produced by algal commensals^49^. However, the poor annotations in CCMP470 of osmolyte producing pathways of diatoms did not allow us to evaluate which organosulfur compounds this diatom is able to synthesize, in order to be able to infer metabolic exchanges.

## Concluding remarks

In this study, we used morphological and genomic approaches to demonstrate that the strain CCMP470, previously annotated as belonging to the radial centric genus *Leptocylindrus*, is in fact an araphid pennate diatom *Plagiostriata* within the staurosiroid clade. Staurosiroid diatoms are characterized by their small cell size, with an apical axis length mostly below 20 μm^16^, and absence of the well-developed labiate processes that are typical of many araphid pennates. *Plagiostriata* is a small genus currently containing two marine species, *P. goreensis* and *P. baltica*. These share morphological characteristics such as apical slits on the valve and the presence of a highly reduced labiate process located along the sternum at the centre of the valve. The latter feature is also seen in CCMP470, leading us to provisionally allocate it into the genus, as also substantiated by the robust support of the phylogenetic position of CCMP470 in the *Plagiostriata* lineage (Fig. 4A).

While staurosiroid lineage diatoms are only poorly studied because of their small cell size, several genera within this group are diverse and often abundant in both marine and freshwater environments, and in planktonic as well as benthic assemblages^4^. Examples include *Staurosira* (and its close relatives), *Opephora* and *Nanofrustulum*. Although the hidden diversity of the staurosiroids has become clearer in recent years (see^16^ and refs therein), no whole genome had been reported from this group of diatoms prior to our study.

The strain CCMP470 has been used in various experiments, e.g.^43,50^, which refer to the diatom as *Leptocylindrus danicus*, or simply as a representative of centric diatoms. Conclusions resting on the taxonomic placement of CCMP470 may therefore need to be reconsidered in light of our results. Since long term culturing can induce valve deformity (e.g.^51^), and CCMP470 was isolated in 1972 and has resided in culture since then, at present we refrain from describing a new species until the discovery of further strains of this “taxon” will allow to illustrate its actual morphology and the range of its variations based on fresh or recently isolated cells. More generally, our work also highlights the problem of identifying some small diatoms, and the risk that strains held in culture collections may be incorrectly annotated. Such identification errors can have implications for subsequent physiological and phylogenetic studies using them.

The draft genome of CCMP470 further identified a bacterial commensal within, which may be one of a number of commensal proteobacterial sequences occurring in other eukaryotic genomes and transcriptomes. It will be necessary to return to environmental samples (e.g., using co-association approaches^52,53^) to identify whether the bacterial symbiont identified in this study also co-occurs with CCMP470 in the wild, or is a post-isolation introduction.

Whole genome or transcriptome sequences are powerful resources to reveal the evolutionary history and encoded functions of organisms of interest. The whole genome sequence of CCMP470 is the first representative of a staurosiroid diatom, and is also the first marine araphid pennate diatom genome available after that of the freshwater species *Synedra acus*^25^. These groups include potentially important contributors in estuarine, coastal and open-ocean assemblages, as well as model systems for bioindustrial cultivation^54,55^, which were previously only represented by one MMETSP transcriptome. CCMP470 may emerge as a useful model system for this clade as it can be kept clonally for long time periods: it has survived in culture since 1972 with no obvious size changes (presumably consistent with changes in physiology), sexual reproduction, or auxosporulation. In addition, the co-sequenced bacterial commensal species may provide functional insights into the *in situ* biological roles of Rhodobacterales, an order of bacteria frequently found in association with marine phytoplankton, and potentially also present in other published algal genome and transcriptome libraries.

## Materials and Methods

### Microscopy

Strain CCMP470 was obtained from the NCMA culture collection. Microscopic observations on exponentially growing cultures of CCMP470 were undertaken with light microscopy (LM; Zeiss Axiophot microscope, Carl Zeiss, Oberkochen, Germany, equipped with phase contrast and bright-field optics and a Zeiss Axiocam digital camera), scanning electron microscopy (SEM; JEOL JSM-6500F, JEOL-USA, Peabody, MA, USA), and transmission electron microscopy (TEM; LEO 912AB, LEO, Oberkochen, Germany). Samples were critical-point-dried (Polaron E3000 Series II, Thermo Scientific, Milan, Italy) for SEM or acid cleaned with 1:1:4, sample: HNO_3_: H_2_SO_4_, sputter coated with gold-palladium using a SC7640 Auto/Manual High Resolution Sputter Coater (Polaron Thermo Scientific, Milan, Italy), and mounted on aluminum stub for SEM or on formvar-coated grids for TEM.

### Draft genome assembly

DNA extraction and PCR amplification were performed as described by^10^. The genome was sequenced using a Whole Genome Shotgun strategy for Roche/454 Titanium technologies. Briefly, 15 µg of DNA were sheared to about 3 or 8 kb, end-repaired with the END-it-Repair kit (Epicentre), and ligated to biotinylated loxP adaptors (Roche). After gel size selection of 3 or 8 kb bands and fill-in, 300 ng DNA were circularized by the Cre recombinase and the remaining linear DNA was digested by the Plasmid Safe ATP-dependent DNAse (Epicentre) and exonuclease I. Circular DNA was fragmented by Covaris (Covaris Inc., USA) shearing and biotinylated fragments were immobilized on streptavidin beads. The library was prepared following the Roche/454 protocol. After library quantification by qPCR, emulsion PCRs were performed. The libraries were then loaded on one PTP and pyrosequenced using the GS FLX Titanium Instrument (Roche) according to the manufacturer’s protocol. A total of 2,666,857 reads (795,989,340 bp) were obtained and assembled using Newbler software (version vMapAsmResearch-04/19/2010-patch-08/17/2010) and default parameters. Contigs were separated into those of probable bacterial and of host origin using LCA analysis, as described in^22,23^, and MetaBAT analysis, as described in^21^).

### Functional analyses

Protein prediction was based on NCBI ORFinder (https://www.ncbi.nlm.nih.gov/orffinder/), except that we did not consider start and stop codons within conserved domains. Introns were identified by the generalized mode of GENESCAN^56^ for each one of the ORFs. For functional analyses, GO category annotation was generated using the Pfams found by CLADE^28^ and refined by GO consortium PFAM2GO (citation http://current.geneontology.org/ontology/external2go/pfam2go), and compared to equivalent domain annotations for the diatoms *Phaeodactylum tricornutum, Thalassiosira pseudonana, T. oceanica* and *Fragilariopsis cylindrus*^24,30–32^. The ModelSEED framework was used to automatically produce annotations and draft genome-scale metabolic models for the diatom and associated bacterial partner^44^. The input files used were the parsed peptide sequences for the bacteria and diatom (Table S1).

### Phylogenetic analysis

#### Diatom phylogeny

A concatenated alignment of conserved diatom BUSCOs was assembled using the eukaryote-odb9 library, following previous methodology, for an assembled set of diatom genomes and MMETSP transcriptomes^33^, along with the assembled genome sequences of the eustigmatophytes *Nannochloropsis gaditana* and *N. oceanica*, the phaeophytes *Ectocarpus siliculosus* and *Cladosiphon okamuranus*, and the pelagophyte *Aureococcus anophagefferens*^57–60^. A total of 35 BUSCOs were selected for concatenation. These BUSCOs had the highest frequency of complete, single-copy coverage across all diatom libraries, excluding sequences that resolve with non-diatom outgroups in single-gene trees (Fig. 3, Table S2).

Furthermore, three gene markers, SSU, *rbc*L, and *psb*C, were concatenated to make a single alignment. Sequences amplified from CCMP470 and *Plagiostriata goreensis*, along with 6 species of leptocylindrids sequenced by^11^, were manually appended to the alignment by^14^ who performed a phylogenetic analysis of araphid diatoms with special emphasis on staurosiroids, and included a wide range of diatom lineages, with *Bolidomonas* as an outgroup. Furthermore, additional staurosiroid sequences were also appended after^14,16^ who newly sequenced further staurosiroids with the description of new *Plagiostriata* species, *P. baltica*. The length of the final datasets was 4,221 bp (1,616 bp for SSU, 1,473 bp for rbcL and 1,132 bp for psbC). RAxML 8.2.0^61^ was used for ML analyses with the GTRGAMMAI model, with partitions for each codon position for protein-coding gene for which gamma correction values and a proportion of invariable sites were obtained automatically by the program. For each dataset the best scoring ML tree was obtained with 200 replicates of hill-climbing searches; we performed 1,000 bootstrap analyses.

### Identification of bacterial sequences in algal transcriptomes

A set of 27 single-copy alpha-proteobacterial marker genes identified from a previous study^40^ were searched in all published alpha-proteobacterial genomes in NCBI, and the top 500 hits obtained to the query HMM sequences were extracted. A similar search was performed against a composite set of all non-alphaproteobacterial prokaryotic libraries, extracting the top five hits; and from all previously published algal genomes, MMETSP and 1KP transcriptomes, retaining only the best hit from each library. This composite set of sequences was aligned, following a previously defined pipeline^33^, and was used to build single-gene RAxML trees with the PROT + GAMMA + JTT substitution model and 100 bootstrap replicates. Sequences that resolved within a paraphyletic group of alpha-proteobacteria and eukaryotes, to the exclusion of the five best non-alphaproteobacterial prokaryotes, were inferred to be of probable alpha-proteobacterial origin (Table S2 for sequence alignments).

### Identification of lineage-specific and horizontally acquired genes

Reciprocal BLAST best hit searches were performed for each genome against 144 taxonomic sub-categories of a combined library of nr, genomic, MMETSP and 1kp transcriptome sequences, as per previous studies^e.g.^ ^33^. To identify possible horizontal gene transfer events for each gene, the reciprocal BLAST best hits for each gene were assembled into a composite reference library, which was searched using BLASTp by using the gene sequence. Each gene was assigned a particular evolutionary affinity if the first two non-redundant hits, as ranked by e-value, corresponded to different taxonomic sub-groups of the particular lineage^33^. Where conflicting patterns of BLAST hits were obtained, the deeper phylogenetic origins of each hit were used to infer deeper unplaced positions for each gene: for example, a gene would be identified as being of diatom origin if the first two non-redundant hits corresponded to different diatom groups; of stramenopile origin if the first two hits corresponded to a diatom and a non-diatom stramenopile group; and of eukaryotic origin if the first two hits corresponded to a diatom and a non-stramenopile eukaryote.

Single-gene trees were generated for candidate HGT events using a previously defined pipeline^33^. Briefly, the query sequence, the single best hits from each of the 144 prokaryotic and eukaryotic categories defined as above, and the 50 additional best hits identified through a BLASTp search of the query sequence against nr were aligned iteratively using MAFFT v 5.0^62^, MUSCLE v 8.0^63^ and the in-built alignment function in GeneIOUS v 4.76^64^, under default settings. After each round of alignment, poorly aligned sequences were manually identified and removed; clusters for which the query sequences failed to align with the nr best hits were excluded from subsequent steps within the pipeline. Each curated alignment was trimmed manually at the N- and C-termini to exclude regions of sequence upstream of the first residue and downstream of the last residue with>70% identities, and with trimAl with setting –gt 0.5^65^. Single-gene trees were generated using RAxML with the PROTGAMMAJTT substitution matrix, and 100 rounds of bootstrapping, and were inspected for concordance between the CCMP470 sister-group and the predicted closest relative identified by BLAST top hit analysis^23,61^.

## Supplementary information

Supplementary Table S2

Supplementary Table S3

Supplementary Table S4

Supplementary Figure Legends

Supplementary Figures

Supplementary Table S1

Dataset S1

Dataset S2
