# Supplementary material for: Genome-enabled phylogenetic and functional reconstruction of an araphid pennate diatom Plagiostriata sp. CCMP470, previously assigned as a radial centric diatom, and its bacterial commensal
Source: Sci Rep. 2020 Jun 10;10:9449. doi: 10.1038/s41598-020-65941-x (PMC7287063; doi:10.1038/s41598-020-65941-x)
Supplement: Supplementary file 4 — Supplementary Figure Legends [file 41598_2020_65941_MOESM4_ESM.docx]

**Genome-enabled phylogenetic and functional reconstruction of an araphid pennate diatom *Plagiostriata* sp. CCMP470, previously assigned as a radial centric diatom, and its bacterial commensal.**

Shinya Sato^1,13^, Deepak Nanjappa^2,11,13^, Richard G. Dorrell^3,13^, Fabio Rocha Jimenez Vieira^3,13^, Elena Kazamia^3^, Leila Tirichine^3,12^, Alaguraj Veluchamy^3^, Roland Heilig^4^, Jean-Marc Aury^4^, Olivier Jaillon^4^, Patrick Wincker^4^, Zoltan Fussy^5,6^, Miroslav Obornik^5,7^, Sergio A. Muñoz-Gómez^8^, David G. Mann^9,10^, Chris Bowler^3*^, Adriana Zingone^2^

^1^ Fukui Prefectural University, Fukui 917-0003, Japan

^2^ Stazione Zoologica Anton Dohrn, Villa Comunale, 80121 Napoli, Italy

^3^ Institut de Biologie de l'ENS (IBENS), Département de biologie, École normale supérieure, CNRS, INSERM, Université PSL, 75005 Paris, France

^4^ Génomique Métabolique, Genoscope, Institut Francois Jacob, CEA, CNRS, Univ Evry, Université Paris-Saclay, 91057 Evry, France

^5^ Biology Centre CAS, Institute of Parasitology, Ceske Budejovice, Czech Republic

^6^ Charles University, Faculty of Science – BIOCEV, Prague, Czech Republic

^7^ University of South Bohemia, Faculty of Science, Ceske Budejovice, Czech Republic

^8^ Centre for Comparative Genomics and Evolutionary Bioinformatics, Department of Biochemistry and Molecular Biology, Dalhousie University, Halifax, Canada

^9^ Royal Botanic Garden, Edinburgh EH3 5LR, Scotland, UK

^10^ Institute for Food and Agricultural Research and Technology (IRTA), E-43540 Sant Carles de la Ràpita, Catalunya, Spain

^11^ Present address: Stony Brook University, School of Marine and Atmospheric Sciences, Southampton, New York, USA

^12^ Present address: Université de Nantes, CNRS, UFIP, UMR 6286, F-44000 Nantes, France

^13^ These authors contributed equally: Shinya Sato, Deepak Nanjappa, Richard G. Dorrell and Fabio Rocha Jimenez Vieira.

*email: [cbowler@biologie.ens.fr](mailto:cbowler@biologie.ens.fr)

Running title: Phylogeny of CCMP470

**Supplementary Figure Legends**

**Figure S1. Characteristics of CCMP470 metagenome. A.** Last common ancestor reconstruction of the entire CCMP470 genome, using methodology adapted from^(22,23)^. **B**. BUSCO coverage of different diatom genomes using plant and protist reference models

**Figure S2. Diatom functional characterization based on Pfams from CCMP470. A.** Distribution of GO categories with cellular functions that are enriched in the CCMP470 genome, as identified by Pearson significance test (P < 0.05). Categories are shaded by organelle of origin: blue (nucleus); green (respiratory organelles); brown/ red (secretory organelles); purple (cytoplasm/ other). **B**. Pfams either present in at least 5 copies in CCMP470 and absent from other diatom genomes; or absent from CCMP470 and present in at least five copies in other diatom genomes.

**Figure S3. Genes specific to araphid diatoms.** **A.** Evolutionary distribution of five genes in the CCMP470 exclusively identifiable in other araphid pennate diatom libraries, as inferred by BLASTp search with threshold evalue 10^-05^. **B.** Exemplar alignment of the CCMP470 g4562 peptide sequence, and that of orthologues from *Astrosyne, Striatella* and *Staurosira* MMETSP libraries, revealing the presence of large numbers of conserved residues.

**Figure S4. Characterization of g7872 gene from CCMP470. A**. Domain map of the CCMP470 g7872 gene, which consists of an N-terminal carbonic anhydrase and C-terminal Hcf164/ thioredoxin domain, linked by five alpha helical and six beta sheet domains of no apparent structural similarity to known conserved domains. **B.** Consensus Bayesian (MrBayes, Jones, WAG) tree of a 47 taxa x 188 aa alignment of the N-terminal region, and a 28 taxa x 138 aa alignment of the C-terminal region of protein g7872. The N-terminal region is of apparent Verrumicrobial origin, whereas the C-terminal region is of clear diatom origin. Dark circles denote nodes where MrBayes PP=1 in all three substitution matrices, and gray circles denote PP>0.8 in two or three of the substitution matrices GRT, Jones and WAG. Where values are shown, these are support values for the node with MrBayes stastics shown on the top and RAxML boostrap values at the bottom, each performed with 3 substitution matrices.

**Figure S5. Characterization of bacterial component of CCMP470 metagenome. A.** Evolutionary origins of each bacterial gene, assessed by BLAST rank analysis. **B.** Numbers of genes in the CCMP470 symbiont genome for which the closest three evolutionary hits reside within eukaryotic sequence datasets, suggestive of wide-spread symbiotic relationships.

**Table S1. Functional characteristics of the CCMP470 genome.**

**Table S2. Sequences, alignments and tree topologies for the multigene BUSCO tree of CCMP470.**

**Table S3. Reciprocal BLAST best-hit and BLAST top hit rank data for the CCMP470 host and symbiont.**

**Table S4. Alignments and single-gene trees for candidate HGT events in the CCMP470 host and symbiont.**

**Dataset S1. HTML format output of the last common ancestor annotation of the CCMP470 metagenome.**

**Dataset S2. SBML format output of ModelSEED metabolic pathway annotation of the CCMP470 symbiont.**
