# Supplementary material for: Genome-enabled phylogenetic and functional reconstruction of an araphid pennate diatom Plagiostriata sp. CCMP470, previously assigned as a radial centric diatom, and its bacterial commensal
Source: Sci Rep. 2020 Jun 10;10:9449. doi: 10.1038/s41598-020-65941-x (PMC7287063; doi:10.1038/s41598-020-65941-x)
Supplement: Supplementary file 5 — Supplementary Figures [file 41598_2020_65941_MOESM5_ESM.pdf]

Figure S1

A)

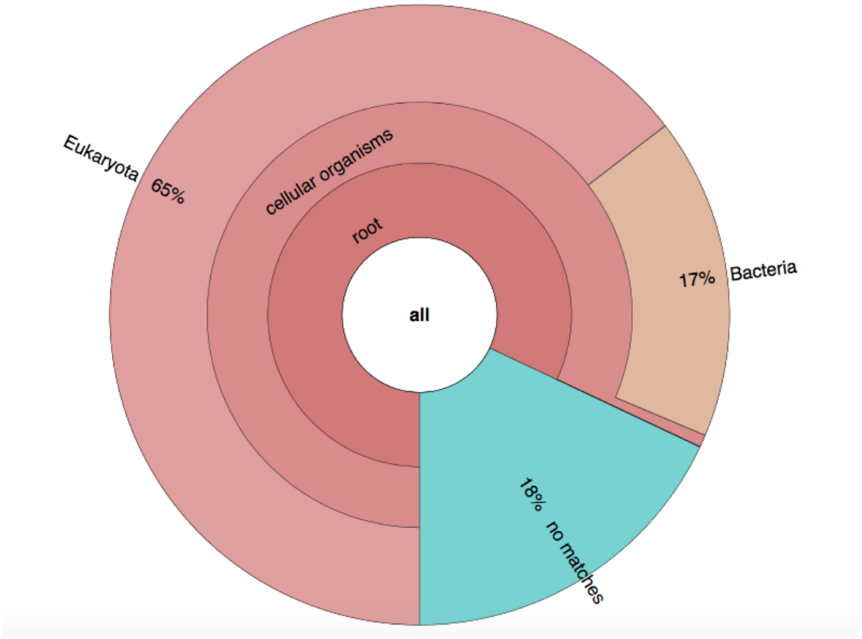
Eukaryota
65%
cellular organisms
root
all
17%
Bacteria
18%
no matches

B)

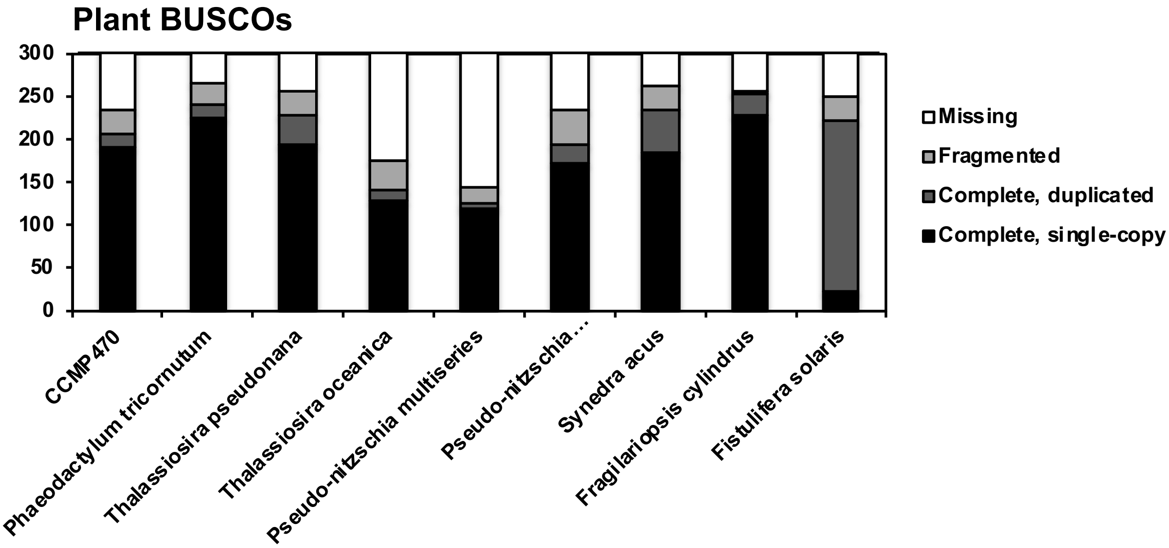
Plant BUSCOs
300
250
200
150
100
50
0
Missing
Fragmented
Complete, duplicated
Complete, single-copy
CCMP470
Phaeodactylum tricornutum
Thalassiosira pseudonana
Thalassiosira oceanica
Pseudo-nitzschia multiseries
Pseudo-nitzschia…
Synedra acus
Fragilariopsis cylindrus
Fistulifera solaris

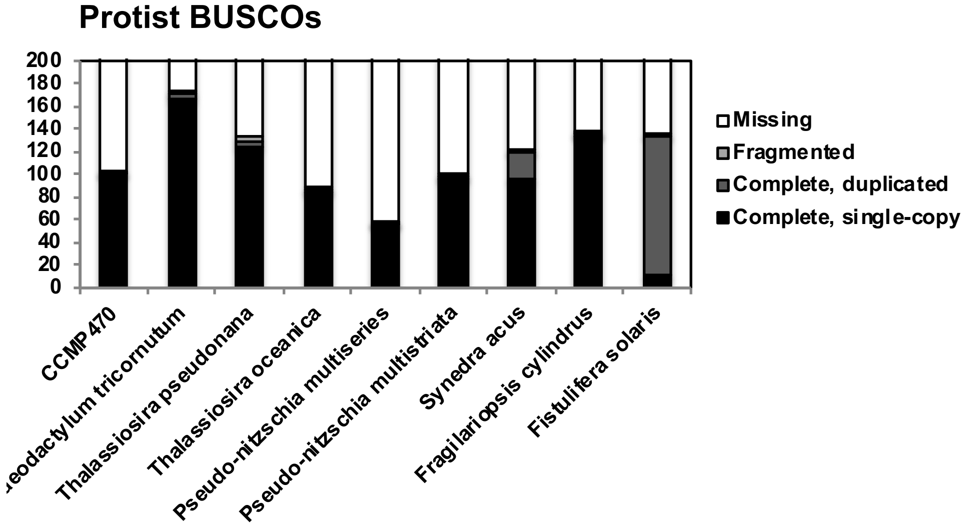
Protist BUSCOs
200
180
160
140
120
100
80
60
40
20
0
Missing
Fragmented
Complete, duplicated
Complete, single-copy
CCMP470
Thalassiosira pseudonana
Thalassiosira oceanica
Pseudo-nitzschia multiseries
Pseudo-nitzschia multistriata
Synedra acus
Fragilariopsis cylindrus
Fistulifera solaris

Figure S2

A)

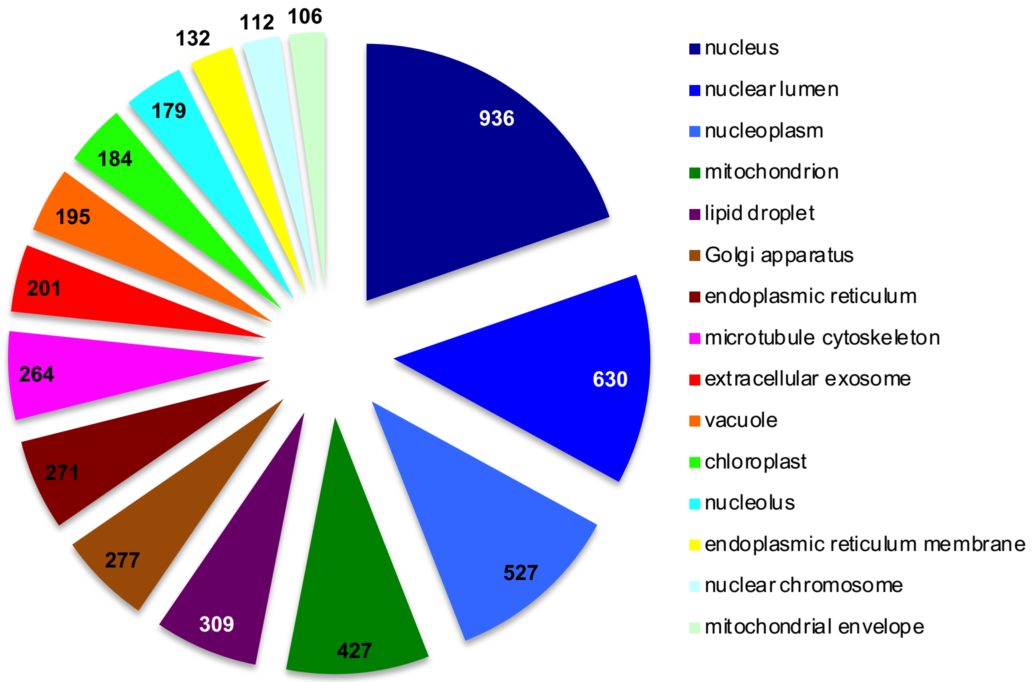

936
630
527
427
309
277
271
264
201
195
184
179
132
112
106
nucleus
nuclear lumen
nucleoplasm
mitochondrion
lipid droplet
Golgi apparatus
endoplasmic reticulum
microtubule cytoskeleton
extracellular exosome
vacuole
chloroplast
nucleolus
endoplasmic reticulum membrane
nuclear chromosome
mitochondrial envelope

B)

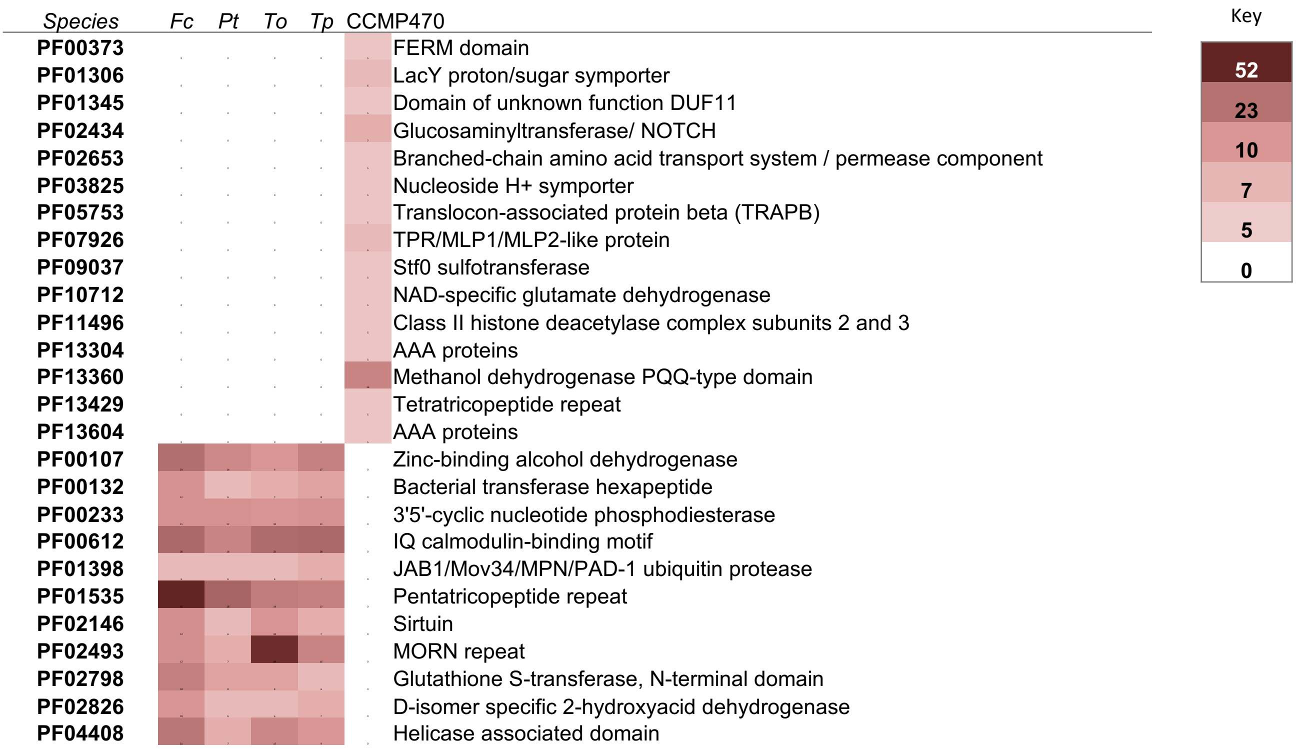

Species Fc Pt To Tp CCMP470
PF00373 FERM domain
PF01306 LacY proton/sugar symporter
PF01345 Domain of unknown function DUF11
PF02434 Glucosaminyltransferase/ NOTCH
PF02653 Branched-chain amino acid transport system / permease component
PF03825 Nucleoside H+ symporter
PF05753 Translocon-associated protein beta (TRAPB)
PF07926 TPR/MLP1/MLP2-like protein
PF09037 Stf0 sulfotransferase
PF10712 NAD-specific glutamate dehydrogenase
PF11496 Class II histone deacetylase complex subunits 2 and 3
PF13304 AAA proteins
PF13360 Methanol dehydrogenase PQQ-type domain
PF13429 Tetratricopeptide repeat
PF13604 AAA proteins
PF00107 Zinc-binding alcohol dehydrogenase
PF00132 Bacterial transferase hexapeptide
PF00233 3'5'-cyclic nucleotide phosphodiesterase
PF00612 IQ calmodulin-binding motif
PF01398 JAB1/Mov34/MPN/PAD-1 ubiquitin protease
PF01535 Pentatricopeptide repeat
PF02146 Sirtuin
PF02493 MORN repeat
PF02798 Glutathione S-transferase, N-terminal domain
PF02826 D-isomer specific 2-hydroxyacid dehydrogenase
PF04408 Helicase associated domain
Key
52
23
10
7
5
0

Figure S3

A)

Gene	*Staurosira complex*	*Grammatophora oceanica*	*Synedra RCC2510*	*Astrosyne radiata*	*Striatella unipunctata*
g8671	1				
g8195	3				
g7750	1	1			
g7216			1		
g4562	1			1	1

B)

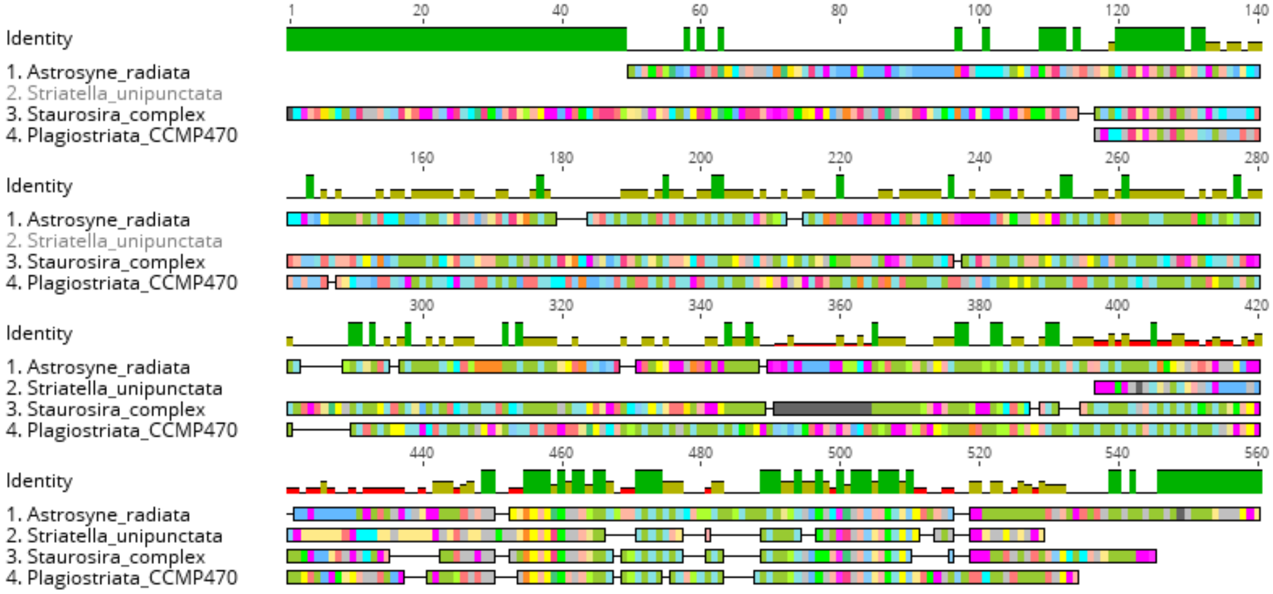

Figure S4

A)

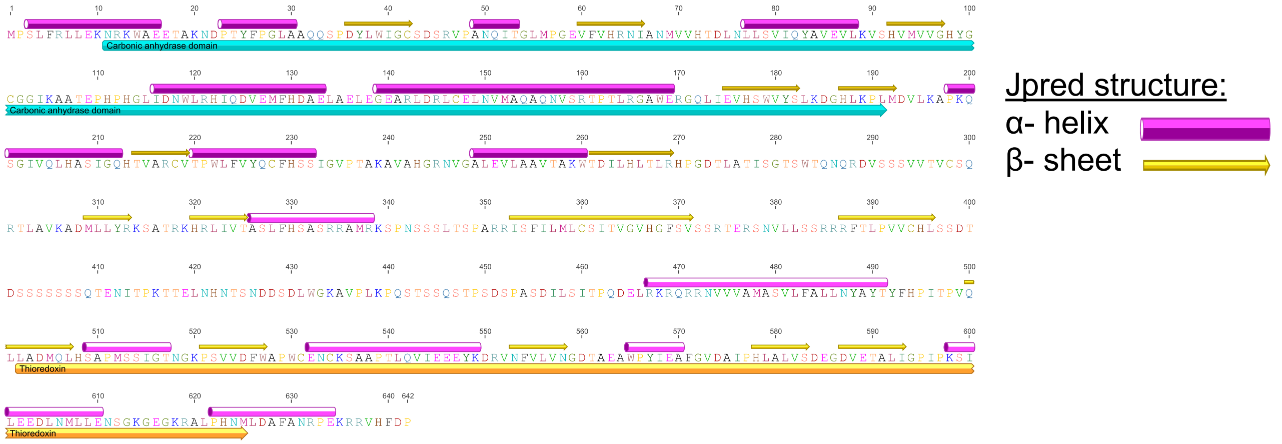

B)

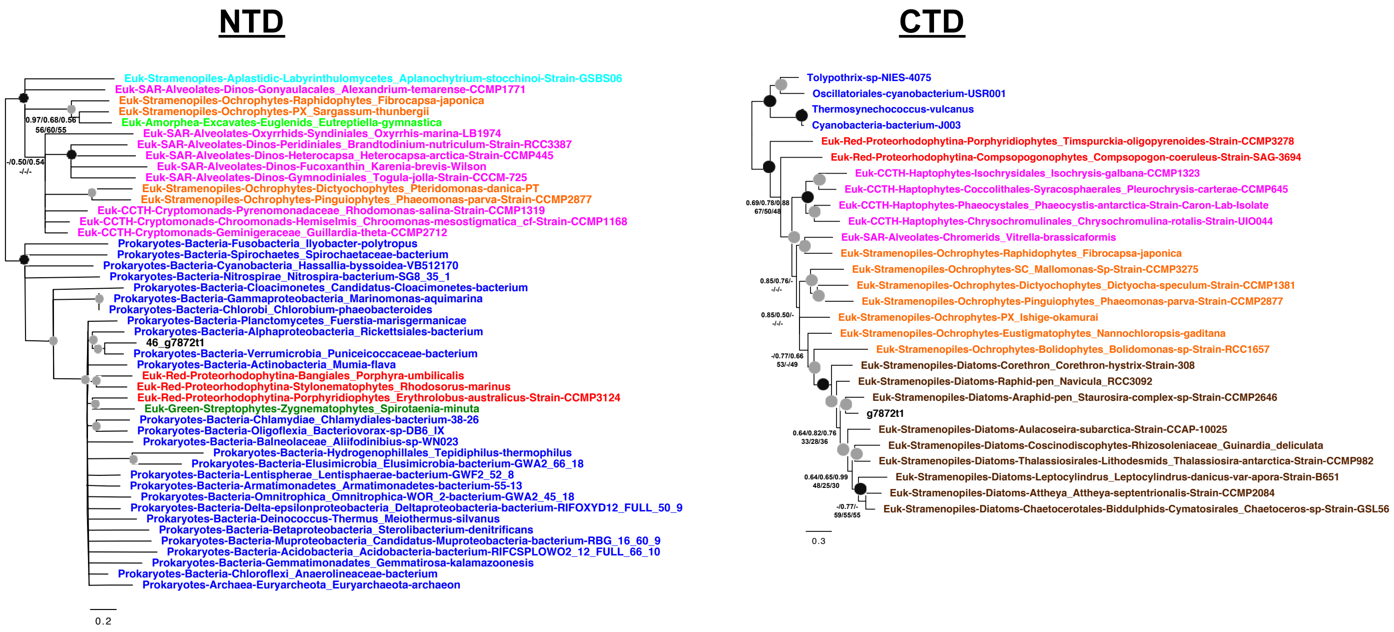

Key

- CCMP470
- Prokaryotes
- Diatoms
- Other ochrophytes
- Other algae with plastids of secondary red origin
- Algae with plastids of secondary green origin
- Aplastidic SAR clade members
- Red algae
- Green algae

Figure S5

A)

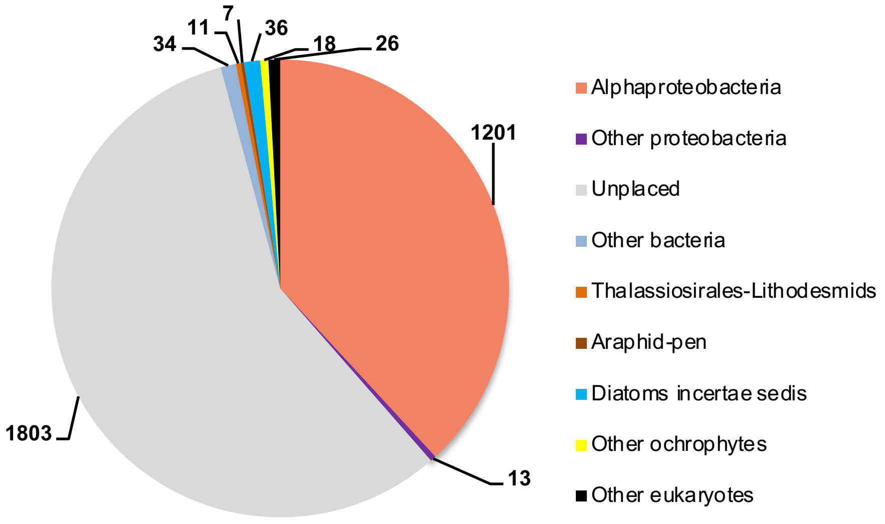

34
11
7
36
18
26
1201
1803
13
Alphaproteobacteria
Other proteobacteria
Unplaced
Other bacteria
Thalassiosirales-Lithodesmids
Araphid-pen
Diatoms incertae sedis
Other ochrophytes
Other eukaryotes

B)

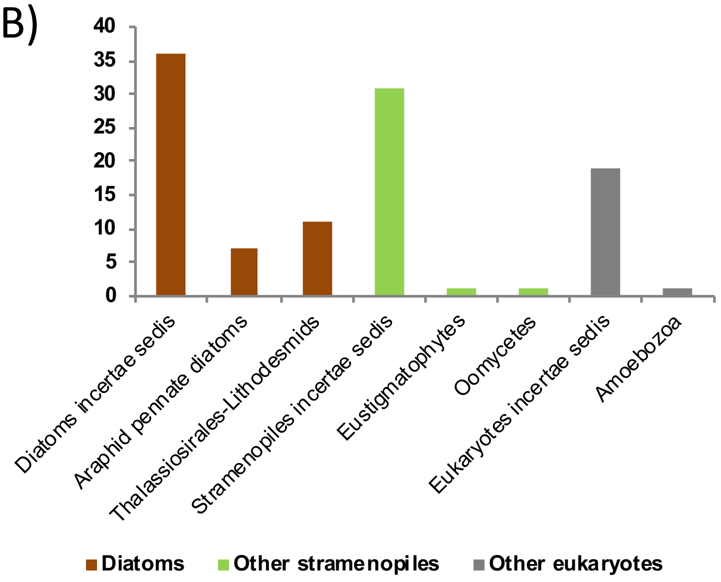

40
35
30
25
20
15
10
5
0
Diatoms incertae sedis
Araphid pennate diatoms
Thalassiosirales-Lithodesmids
Stramenopiles incertae sedis
Eustigmatophytes
Oomycetes
Eukaryotes incertae sedis
Amoebozoa
Diatoms
Other stramenopiles
Other eukaryotes
