# Supplementary material for: Genome-enabled phylogenetic and functional reconstruction of an araphid pennate diatom Plagiostriata sp. CCMP470, previously assigned as a radial centric diatom, and its bacterial commensal
Source: Sci Rep. 2020 Jun 10;10:9449. doi: 10.1038/s41598-020-65941-x (PMC7287063; doi:10.1038/s41598-020-65941-x)
Supplement: Supplementary file 7 — Dataset S1 [file 41598_2020_65941_MOESM7_ESM.html]

Javascript must be enabled to view this page.

magnitude
magnitudeUnassigned

ccmp470 contigs

7732

6341

2

49
6339

625
4992

4

4

4

4

4

4

3

1

1
9

1

1

1

1

1

1

1

1

2

1

1

1

1

1

1

2

1

1

1

1

1

1

2

2

2

2

2

8

3

3

3

3

3

3

3

3

3

3

3

5

1

1

1

1

1

4

3

3

1

2

1

1

1

9

2

2

2

2

2

4

3

3

3

3

1

1

1

1

2

2

2

2

2

1

1

1

1

1

1

1

1

1

1

1

1

1
96

16

16

16

16

16

16

16

78
3

1

1

1

1

3

3

3
1

1

1

1

1

1

69

68
1

18

18

49

49

1

1

1

2

1

1

1

1

1

1

1

1

1

1

1

1

1

1

4225
74

1

1

1

5

5

2

2

3

3

1

1

1

1

1

2

1

1

1

1

1

1417
4142

13
145

29
1

13

13

13

13

12

12

7

7

5

5

3

3

3

3

45

45

45
2

14

14

29

29

58

58

3
58

36

36

19

19

389

48
389

5

5

109

109
19

14

14

10

10

34

34

34

22
12

2

2

2

2

2

10

8

2

202
11

2
78

69

69

4

4

3

3

54

54

54

1

1

1

25

25
22

3

33

33

33

25

25

25

25

1873

1873
320

68

5
68

7

7

56
12

18

26

1242

1242
169

173

173

205

205

624

71

71

54

54

54

101

101
4

48

48

49

49

88

57

57

57

31

31

31

318
23

110
3

96

44

44

44

52

28
52

2

13

5

2

2

11

11

11

11

1

1

1

1

1

69

2

2

2

2

44

44

44

23

23

23

23

104

15
104

2
75

11
67

1

1

4

4

34

5

4

2

2

2

3

4

4

2

2

13

13
5

1

1

3

3

3

1

1

1

11

11

11

5
1

4

1

1

3

3

2

2

7

4

1

1

1

1

3

3

2

1

3

3

3

3

2

2

2

2

2

4

4

4

2

1

1

1

1

1

1

1

1

1

1

1

1

1

1

1

1

2

1

1

1

1

1

1

1

1

1

1

1

1

1

2

1

1

1

1

1

1

1298
45

2

2

2

2

2

2

2

2

13
3

1

1

1

1

1

3

2

2

2

2

2

1

1

1

1

1

3

3
2

1

3

1

1

1

1

1

1

2

2

1

1

1

1

1

1

1235
60

4
20

5

5

3

2

4
1

1

1

1

1

2

2

2

2
1

1

1

1

1

1

1

1

1

1

1

1

1

1

1

1

1

1

1

1

1

1

1

1

1

1

1

1

121
1150

2
24

1

1

5

5
4

1

1

2

2

2

1
10

8

2
7

2

2

1

1

3

3

1

1

1

1

1

1

1

1

2

1

1

1

1

1

1

2

1
2

1

1

2

2

2

2

2

53
1000

248
947

671
7

660

2

1

1

1

2

2

2
6

4

1

1

1

1

1

1

1

1

6

2

2

2

2

2

1

1

1

1

1
4

3

3

3

3

3

1

1

1

1

1

3
2

1

1

1

2

1

1

1

1

1

1

1

1

1

1

3

3

2
1

1

1

1

1

1

1

3

3

3

1

1

1

1

1

1

2

2

2

1391
